# Prehabilitation for Bariatric Surgery: A Randomized, Controlled Trial Protocol and Pilot Study

**DOI:** 10.3390/nu13092903

**Published:** 2021-08-24

**Authors:** Yaiza García-Delgado, María José López-Madrazo-Hernández, Dácil Alvarado-Martel, Guillermo Miranda-Calderín, Arantza Ugarte-Lopetegui, Raúl Alberto González-Medina, Alba Hernández-Lázaro, Garlene Zamora, Nuria Pérez-Martín, Rosa María Sánchez-Hernández, Adriana Ibarra-González, Mónica Bengoa-Dolón, Carmen Teresa Mendoza-Vega, Svein Mikael Appelvik-González, Yurena Caballero-Díaz, Juan Ramón Hernández-Hernández, Ana María Wägner

**Affiliations:** 1 Department of Endocrinology and Nutrition, Complejo Hospitalario Universitario Insular-Materno Infantil, 35016 Gran Canaria, Spain; marialmendocrino@gmail.com (M.J.L.-M.-H.); nuriapm@hotmail.com (N.P.-M.); rosamariasanher@gmail.com (R.M.S.-H.); aibarraggg@gmail.com (A.I.-G.); 2 Instituto Universitario de Investigaciones Biomédicas y Sanitarias, Universidad de Las Palmas de Gran Canaria, Las Palmas de Gran Canaria, 35001 Las Palmas, Spain; dacil.alvarado@ulpgc.es (D.A.-M.); gbzz@hotmail.com (G.Z.); 3 Department of Rehabilitation and Physical Medicine, Complejo Hospitalario Universitario Insular-Materno Infantil, 35016 Gran Canaria, Spain; gmirandacalderin@gmail.com (G.M.-C.); augarte67@gmail.com (A.U.-L.); cmenveg@gobiernodecanarias.org (C.T.M.-V.); sappgon@gobiernodecanarias.org (S.M.A.-G.); 4 Internal Medicine Nursing 8th North Wing, Complejo Hospitalario Universitario Insular-Materno Infantil, 35016 Gran Canaria, Spain; mooguay@hotmail.com; 5 Department of Endocrinology and Nutrition, Hospital Universitario Dr. Negrín, 35010 Gran Canaria, Spain; albahdezlazaro@gmail.com; 6 Department of Pneumology, Complejo Hospitalario Universitario Insular-Materno Infantil, 35016 Gran Canaria, Spain; mobendo@hotmail.com; 7 Department of General and Digestive Surgery, Complejo Hospitalario Universitario Insular-Materno Infantil, 35016 Gran Canaria, Spain; ycabdiac@gobiernodecanarias.org (Y.C.-D.); jurahh@yahoo.es (J.R.H.-H.)

**Keywords:** prehabilitation, bariatric surgery, preoperative exercise, respiratory training, obesity

## Abstract

Bariatric surgery is the most efficacious treatment for obesity, though it is not free from complications. Preoperative conditioning has proved beneficial in various clinical contexts, but the evidence is scarce on the role of prehabilitation in bariatric surgery. We describe the protocol and pilot study of a randomized (ratio 1:1), parallel, controlled trial assessing the effect of a physical conditioning and respiratory muscle training programme, added to a standard 8-week group intervention based on therapeutical education and cognitive-behavioural therapy, in patients awaiting bariatric surgery. The primary outcome is preoperative weight-loss. Secondary outcomes include associated comorbidity, eating behaviour, physical activity, quality of life, and short-term postoperative complications. A pilot sample of 15 participants has been randomized to the intervention or control groups and their baseline features and results are described. Only 5 patients completed the group programme and returned for assessment. Measures to improve adherence will be implemented and once the COVID-19 pandemic allows, the clinical trial will start. This is the first randomized, clinical trial assessing the effect of physical and respiratory prehabilitation, added to standard group education and cognitive-behavioural intervention in obese patients on the waiting list for bariatric surgery. Clinical Trial Registration: NCT0404636.

## 1. Introduction

Obesity is a global public health issue, associated with high morbimortality and health costs. Bariatric surgery is the most effective treatment for obesity and its complications [1,2,3,4,5,6]. The remission of type 2 diabetes is seen in 23–60% of patients, depending on the type of procedure, as well as the severity and duration of the disease [7,8]. Bariatric surgery can also prevent 82% of new-onset diabetes [1], improve glycaemic control [9,10], and reduce diabetes complications more effectively that non-surgical treatments [4,5,9,10]. Hypertension improves within the first postoperative month and this improvement is long-lasting [2,11] and the need for lipid-lowering drugs and continuous positive air pressure therapy during sleep are also reduced by 82% and 70–90%, respectively [3]. Furthermore, after bariatric surgery, obese patients have lower cardiovascular, cancer, and all-cause mortality than unoperated peers [5,8,12]. Nevertheless, although surgery is the most effective weight-loss measure, obese people are at high risk for surgical complications. Thus, success depends not only on the chosen surgical procedure but also on the patient’s preparedness for surgery. Indeed, psychosocial-behavioural assessment, as well as a multidisciplinary intervention on diet, physical activity, and behaviour change are recommended [13].

Preoperative weight loss is associated with a reduction in surgical complications, intervention complexity, and hospital length of stay [6,14,15,16,17,18,19]. Tested interventions include lifestyle changes, nutritional education, physical activity, cognitive-behavioural therapy, and pharmacological treatment. In a systematic review and meta-analysis, pre-operative weight loss appeared to be associated with greater post-operative weight loss [20], an effect which is especially evident in patients with a BMI above 45 kg/m^2^ [21].

In 2016, the Enhanced Recovery After Surgery (ERAS) Society published its guidelines for bariatric surgery [22], which included preoperative weight-loss, smoking, and alcohol cessation and preoperative instructions for increased adherence, quick recovery and shorter hospital stay. In addition, the guidelines now include “prehabilitation”, preoperative physical conditioning to increase functional capacity and prepare the patient for the metabolic stress associated with surgery. In abdominal oncological surgery, orthopaedic and cardiopulmonary surgery, prehabilitation has led to improved postoperative respiratory and muscular functional capacity [23,24,25,26,27,28,29,30,31,32,33,34,35,36,37]. There is less evidence of harder outcomes, such as complications and length of hospital stay [38]. In obese patients, preoperative physical activity is associated with a reduction in cardiometabolic risk, premature mortality, and postoperative complications [39,40], but specific interventions before bariatric surgery are scarce. Hence, the recommendation of prehabilitation by ERAS in this context is not strong, though it is described as promising [22]. International guidelines currently recommend specialised dietary advice by a dietitian trained in long-term behaviour changes, both for the preoperative and the postoperative periods [13,41]. A few studies have shown improved postoperative pulmonary capacity and resistance following prehabilitation with the training of inspiratory muscles [42,43,44], which might lead to fewer respiratory complications.

In the present report, we describe the study protocol and the pilot survey for a randomized controlled trial assessing the effect of physical conditioning and inspiratory muscle training (prehabilitation) in people awaiting bariatric surgery, on preoperative weight-loss, comorbidities, and postoperative complications.

## 2. Materials and Methods

### 2.1. Study Design

This is a randomized, parallel, controlled trial assessing the effects of a 16-week programme including physical activity and a minimal respiratory physiotherapeutic intervention on pre-surgical weight-loss in patients awaiting bariatric surgery.

Eligible patients are randomized to one of two treatment groups: a standard, multidisciplinary treatment (control), consisting of an education programme and cognitive-behavioural intervention or the same treatment in addition to physical activity and respiratory muscle training (intervention, i.e., prehabilitation).

Written informed consent is obtained from all participants, and the trial was conducted in accordance with the principles stated in the Declaration of Helsinki and the European Medicines Agency Good Clinical Practice Homogenized Guidelines. The study protocol was approved by the provincial Ethics Committee and registered at ClinicalTrials.gov (NCT04046367) on 6 August 2019.

### 2.2. Study Population

**Figure 1:**
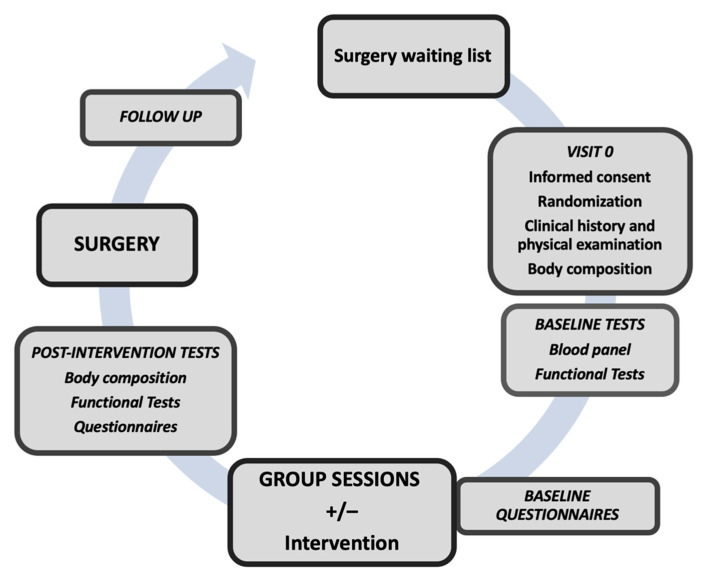
Study flow chart.

All patients on the waiting list for bariatric surgery with obesity grade III–IV selected according to the 2016 Position Statement of the Spanish Society for the Study of Obesity [45], expected to be operated on at least 4 months after inclusion, are potentially eligible and are approached by phone. Those who agree, are scheduled for an appointment at the hospital, where more detail is given about the trial. Participants sign written informed consent before inclusion. The only exclusion criteria are those limiting participation in the programme (according to the investigators’ assessment), such as timing difficulties or linguistic barriers. Figure 1 shows a flowchart of the study.

### 2.3. Randomization and Allocation Masking

Participants are assigned to their treatment group (ratio 1:1) following a computer-generated random sequence. The latter is generated by a lab technician otherwise not involved in the trial. It is transferred to individual cards, which are inserted into opaque, sealed, consecutively numbered envelopes. When the participant signs the consent form and eligibility is confirmed, the first sealed envelope is opened to reveal the treatment group, and the patient is scheduled for the baseline assessment tests and the eight fortnightly sessions.

### 2.4. Intervention Masking

Participants are informed that two different group programmes are compared, but no detail is given about the difference between them. Most outcomes are not expected to be affected by the investigators’ knowledge of the treatment arm. An assessment of functional capacity is performed, whenever possible, by explorers unaware of the participant’s assigned treatment.

### 2.5. Standard Treatment

The standard treatment (control group) consists of a structured, educational, and behavioural programme of eight 2-h, fortnightly sessions, followed by 1-h monthly sessions until surgery. Table 1 describes the contents of the sessions.

At the first session, all participants are given the contents of the education programme in writing and are asked (and trained) to start recording their physical activity by means of a free, mobile app (*Google Fit*) downloaded in their smartphones or an activity tracker, if they owned one, for motivation and monitoring of adherence. To avoid contamination between treatment arms, the control and intervention groups are scheduled every other week.

### 2.6. Intervention (Prehabilitation)

The intervention group receives the same programme as above, but each 2-h standard session is followed by half an hour specific training, aimed at increasing physical activity, functional capacity, and respiratory muscle strength.

**Figure 2:**
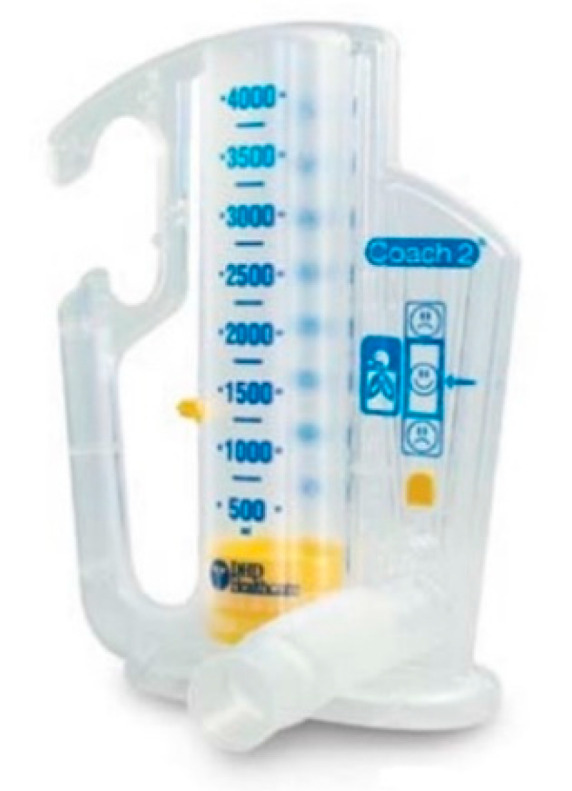
Incentive spirometry device, which provides visual cues to the patients that the desired flow or volume has been achieved.

**Figure 3:**
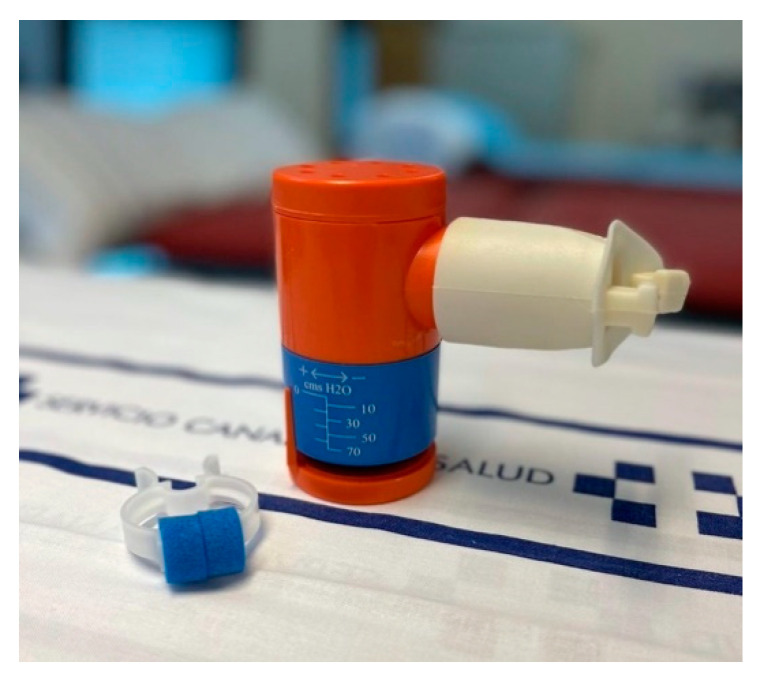
Inspiratory valve used in the training programme.

At the end of the first session, the participants in the intervention group are given a device for incentive spirometry (Figure 2), an inspiratory valve (Figure 3), and elastic non-latex resistance bands (*TheraBand^®^*, Akron, OH, USA), which are colour-coded according to their resistance (yellow, blue, black). Patients are trained in the use of the devices.

Participants are also given general recommendations on physical activity, such as avoiding high UV exposure, using adequate clothing, and shoes and avoiding exercise when sick. They are advised to walk for at least 30 min daily, and then gradually increase this, according to tolerance. The first and final 5-min periods are used for warm-up and cool down, respectively. Participants are also advised to practice all exercises daily and to record them and bring the registers to the following session.

#### 2.6.1. Physical Conditioning

It consists of four resistance exercises with elastic bands, that are to be performed twice daily, for 15–20 min per time, until the following scheduled session. Participants receive spoken and written instruction, as well as practical demonstrations. Four exercises involving large muscle groups of both the upper and lower extremities are proposed (e.g., Kabat’s diagonal, frontal elevation and aperture of the arms, shoulder elevation and rolling, and squats combined with elbow flexion). The concentric phase of muscle contraction is performed during expiration. Each exercise is repeated 15 times per series, and 2–3 series are planned, with recovery time in between. Low resistance bands are used at the beginning of the programme and are progressively changed to higher resistance bands.

#### 2.6.2. Minimal Preoperative Respiratory Physiotherapy Intervention

- Incentive spirometry: The purpose of incentive spirometry is to facilitate a sustained, maximal inspiration (SMI). An SMI is a slow, deep inspiration from the Functional Residual Capacity up to the total lung capacity, followed by 4–8 s breath-hold, mimicking natural sighing. We use *Coach 2*^®^ (*Portex*^®^, Smith-Medicals, Minneapolis, MN, USA) 500–4000 mL devices which provide visual cues to the patients that the desired flow or volume has been achieved (Figure 2). This serves as a motivation and a goal for the patient to try to meet, by repeating the maximal effort frequently. We recommend four repetitions of this maneuver both standing and sitting. Patients are encouraged to use the device twice daily.
- Respiratory exercises: Participants are taught how to perform specific respiratory exercises (breathing with pursed lips, quick and slow expiratory techniques, in order to manage secretions, and assisted cough with the protection of the surgical wound).
- Inspiratory muscle training: This is performed using an inspiratory valve (0–70 cm H_2_O) (*Orygen Inspiratory Valve*, Forumed S.L., Gerona, Spain) (see Figure 3) [46]. Training starts at 30% of the maximal inspiratory pressure (MIP) with progressive 10 cm H_2_O increments from session to session, according to the participant’s tolerance to the exercises. Patients are advised to repeat these exercises for 10 min twice daily, at 2 min intervals.

### 2.7. Outcomes

The primary outcome is the change in body weight between the start of the programme and the time of surgery.

Secondary outcomes include: changes in body composition, comorbidities, changes in eating behaviours, health-related quality of life, functional capacity, length of hospital stay after surgery and short-term complications of surgery.

### 2.8. Clinical History and Blood Tests

Sociodemographic (age, sex, education level, healthcare reference area, working situation), and clinical variables are recorded. A clinical history, physical examination and basic blood tests are performed. Blood pressure is measured after 5 min in the sitting position with a manual sphygmomanometer, adapted to the size of the patient’s arm. HbA1c is measured by HLPC, standardized against DCCT-IFCC. Glucose, total cholesterol, triglyceride, HDL cholesterol, AST, ALT, GGT, and creatinine are measured by automated, colourimetric methods. LDL cholesterol and glomerular filtration rates are calculated. Drug treatment is obtained from the interview and the electronic prescription.

Dyslipidaemia is defined by an LDL cholesterol above 160 mg/dL, triglyceride above 200 mg/dL or lipid-lowering drug treatment. Diabetes is defined by the American Diabetes Association criteria [47] or the need for glucose-lowering treatment. Hypertension is defined by blood pressure ≥140/90 mmHg or blood-pressure-lowering treatment. In women, hirsutism is defined by a score >8 points on the Ferriman–Gallwey scale. Chronic venous insufficiency is recorded if the diagnosis is present in the patient’s clinical records or is evident at physical examination. Other comorbidities associated with obesity, such as infertility, nonalcoholic fatty liver disease (NAFLD), gastroesophageal reflux or hiatal hernia, bronchial asthma, function limiting osteoarthritis, or intracranial hypertension are recorded if a previous diagnosis has been made.

### 2.9. Body Composition

Body composition is assessed at the baseline visit. Weight is assessed with a calibrated bioimpedance (*Tanita BC 420 MA III*, Tokyo, Japan) to the nearest 0.1 kg, and height, using a stadiometer to the nearest 0.5 cm, without shoes and with light clothing. Body Mass Index (BMI) is then calculated as weight (in kg) divided by height (in meters)^2^. Body composition is analyzed through Vectorial Bioimpedance by tetrapolar impedance plethysmography using an electric alternating current flux of 400 µA at an operating single sinusoidal frequency of 50 kHz (*Nutrilab™*, Akern, Firenze, Italy in resting conditions. Standard whole-body measurements are performed according to the manufacturer’s guidelines on the right side of the body with the subjects in supine position with their arms and legs abducted, and the skin cleaned to ensure good contact. The thighs are checked not to be in contact with each other and the arms not to be touching the sides of the body. Electrodes are placed on a line between the radial and ulnar styloid processes on the dorsum of the wrist and on a line between the medial and lateral malleoli on the dorsum of the ipsilateral foot, overlying the head of the third metacarpal on the dorsum of the hand and the third metatarsal on the dorsum of the foot. Total body water, fat-free mass, fat-free mass percentage, fat mass, fat mass percentage and body cell mass are determined according to the formulas of Kotler et al. [48], and bioelectrical parameters of resistance, reactance, and phase angle are registered.

### 2.10. Questionnaires

A series of self-completed questionnaires are completed before the first group session and after the last one. General health-related quality of life is assessed using the Spanish version of EuroQol-5D-5L (EQ-5D-5L) [49]. Dietary habits are explored using the Adherence to the Mediterranean Diet Adherence Screener (MEDAS) questionnaire [50], eating disorders, by means of the Spanish version of the Eating Disorder Inventory (EDI-3) [51,52], and anxiety and depression, using the Spanish version of the Hospital Anxiety and Depression Scale (HADS) [53,54].

### 2.11. Functional Tests

- Six-minute walk test (6MWT): This is a cheap and easy to perform, submaximal stress test. Its aim is to cover the maximal distance in 6 min, walking on a flat surface, following a standardized protocol [55]. The patient is not coached nor stimulated to walk quickly, and he/she can stop and rest as needed. Blood pressure (beginning and end), heart rate (every minute), oxygen saturation (every minute), and self-perceived exertion (Borg, beginning and end). The percentage of the theoretical 6MWT distance (6MWD) is calculated using Enright’s regression equation [56].
- Handgrip strength (HGS) is measured in both hands with a classical, analogic hand dynamometre (0–90 kg, 0–200 Lb) (*JAMAR™* Hydraulic Hand Dynamometer, Preston, Jackson, Missouri, EEUU) following the protocol described by the American Society of Hand Therapists [57], with the patient seated, back and feet resting, shoulders adducted, elbow at 90°, and forearm and wrist in a neutral position. Three 3-s alternating measurements are performed on each hand and the average is calculated and the dominant hand is registered.
- Pulmonary function tests are performed according to the European Respiratory Society (ERS), American Thoracic Society (ATS) and Spanish Society of Pneumology and Thoracic Surgery (SEPAR) technical standards [58,59], by trained staff with a *MasterScreen*, Jaeger, Germany. Forced spirometry is performed under basal conditions, to assess respiratory capacity, maximal inspiratory pressure, and maximal expiratory pressure are measured at baseline. Plethysmography is used to measure pulmonary volumes, residual volume and functional residual capacity. Pulmonary diffusion (DLCO) is assessed by gas dilution. Baseline arterial gasometry is also performed.
- Obstructive Sleep Apnea (OSA) is assessed during sleep by means of home cardio-respiratory polygraphy (*Somnoscreen™*, Sanro, Madrid, Spain), which is then interpreted manually by trained medical staff at the Sleep Unit, following SEPAR and American Academy of Sleep Medicine guidelines [60,61].

### 2.12. Adherence to the Intervention and Physical Activity

Adherence to the intervention is assessed by the attendance at the sessions. Physical activity is monitored using an activity app (*Google Fit* or the manager of an activity tracker) downloaded on a smartphone, as well as the Spanish short version of the International Physical Activity Questionnaire [62].

### 2.13. Monitoring and Follow-Up

Adverse events are registered and classified according to their intensity, severity and potential association with the intervention. In addition, halfway through the intervention (for session 4), participants with hypertension and diabetes are asked to monitor blood pressure and glucose concentrations, respectively. The registers are assessed by one of the endocrinologists, in order to reduce blood pressure and/or glucose-lowering drugs, when needed. These changes are registered.

### 2.14. Surgery

Information about the date, surgical procedure (including Foucher, staple size and type, gastric pouch size, gastrojejunal anastomosis diameter, common channel length), length of hospital stay, and early (first 30 days) complications are obtained from the participant’s clinical record. Post-operative complications are classified according to the Clavien–Dindo classification [63].

### 2.15. Statistical Analyses

At the time of the design of the protocol, we estimated that, during one year, we would be able to include 80 participants, 40 in each treatment arm, following two 10 person groups (one control and one intervention) at a time. The inclusion of 80 participants, without any loss to follow-up, assuming a standard deviation in weight of 11 kg [16], for a two-tailed *p* < 0.05, would allow a difference between groups, with 80–90% statistical power, of 8 and 7 kg to be detected, respectively. Assuming a standard deviation of 6 kg, as shown by other studies [64], the detectable difference would be reduced to around 4 kg.

A baseline descriptive analysis is performed, and variables are expressed as percentages, mean (SD) or median (interquartile range), as appropriate according to their type and distribution. Treatment groups are compared using chi-squared, Student’s *t* or Mann–Whitney’s *U*. Analyses will be performed by intention to treat. Missing values will be imputed using the last observation carried forward.

## 3. Results

Participants in the pilot study had their first visit between 8 August 2019 and 13 September 2019, and the interventions took place between October 2019 and January 2020. 

Out of the 51 patients on the waiting list, expected to be operated on at least 4 months after recruitment, 19 accepted to participate. The main reasons given for declining were participation in preoperative groups in another centre (on a different island), distance to the training site and schedule problems, mainly due to working hours. None of the patients refused to participate or was excluded due to linguistic barriers. A total of 15 (14 women) attended the first visit and were randomized. Their mean age was 40.0 ± 10.0 years, baseline BMI, 46.7 ± 5.9 kg/m^2^, and waist circumference 134.2 ± 12.0 cm. The most frequent complications of obesity were OSA (54%), osteoarticular disorders (40%), asthma (33.3%), hypertension (33.3%), infertility (35.7%), chronic venous insufficiency (28.6%), diabetes (26.7%), and dyslipidaemia (26.7%). Seven patients were randomized to the intervention group and 8 to the control group. Their baseline features are displayed in Table 2.

The first eight sessions took place between October 2019 and January 2020. Three participants dropped out before even starting the intervention (one travelled abroad, another one said the timing of the sessions was inconvenient, and the third did not give a specific reason). The rest attended between one and six sessions. Only five participants completed follow-up, three in the intervention and two in the control group. Of the ten patients not completing the programme, seven interrupted it due to early surgery. Unlike what was initially planned, because of the low attendance at the sessions, we decided to merge both treatment groups from session 2, since group intervention was not possible otherwise. The control group left after the standard session finished, whereas the intervention group joined the physiotherapist team thereafter.

The five participants completing the follow-up lost an average of 0.54 ± 5.2 kg (Intervention group +1.6 ± 4.3 kg; Control group −3.8 ± 5.7 kg) and the final BMI was 48.1 ± 7.6 kg/m^2^ (Intervention Group 45 ± 7.3 kg/m^2^; Control group 52.69 ± 10.6 kg/m^2^). Given the small sample size, no statistical analysis was performed and no conclusions can be drawn.

## 4. Discussion

To our knowledge, this is the first clinical trial to assess the efficacy of physical conditioning and inspiratory muscle training before bariatric surgery, on preoperative weight-loss, comorbidities and postoperative complications, when added to standard care, based on therapeutic education and cognitive-behavioural therapy.

Although there are studies assessing the effect of physical activity in patients with obesity, there is very little evidence on the effect of a preoperative intervention (prehabilitation). We decided to combine physical activity with specific inspiratory muscle training because a decreased respiratory function is associated with an increased risk of postoperative complications [40,65].

Improved preoperative fitness and weight-loss have been assessed in a few small studies. A 12-week, preoperative programme combining endurance activity and resistance exercises was associated with improved fitness and quality of life [66]. One year post-operatively, the benefits of the intervention on physical fitness were enhanced and weight-loss was also slightly greater in the intervention group [67]. Another trial showed preoperative improvements in weight and functional capacity in both the control and the intervention (endurance and resistance training) groups [64], whereas others show pre-operative benefits on weight-loss, comorbidity, and quality of life [68], and persistent pre-operative and post-operative vigorous physical activity [69], as well as improvements in psychological well-being [70]. Sustained benefits of these interventions would probably have positive, long-term effects on associated comorbidity and on health-related quality of life.

Unlike previous studies, our trial includes specific training of respiratory muscles, which could be of added value and expand the concept of prehabilitation in these patients. In order to test the study design and coordination among departments, potential problems and setbacks a pilot study was performed.

The participants included in the pilot group had features that are representative of the patients treated with bariatric surgery in our centre. Two recent reviews of the patients who had undergone surgery between January 2013 to January 2014 (G Ramírez, MD master thesis, unpublished results) and between January 2016 to December 2017 (D Aguiar, MD master thesis, unpublished results) showed that their mean age was 43.6 ± 10.9 and 43.35 ± 16.81 years, 19.2% and 24.31% were male, and their baseline BMI was 48.1 ± 8.64 kg/m^2^ and 46.48 ± 8.82 kg/m^2^, respectively.

The main limitation of this pilot intervention was the low adherence of participants to the programme, though most of the losses to follow-up were due to surgery itself. Other intervention studies also show poor adherence [27,71,72,73,74]. Recruiting participants was also a challenge, since a significant number of patients lived on another island and group programmes are not routinely used in the care of obese patients. Thus, participants probably experience the programme as an additional burden.

The initial protocol was designed to perform independent sessions for control and intervention groups but, due to low adherence, in order to maintain a group intervention approach, we decided to merge them from session 2. The prehabilitation group attended the physiotherapist after the common intervention and the control group left. Contamination could of course be an issue, but there was no time during the sessions to discuss other than the programmed subjects and the participants in the control group did not have the devices and material used for the performance of the exercises. Indeed, with this design, participants are guaranteed an identical intervention, differing only in prehabilitation itself.

The pilot study has indeed led us to reflect on the possible causes of low acceptance of and adherence to the programme, as well as establishing mechanisms to overcome this. Seventy percent of the participants were operated on before the group intervention was completed. Therefore, coordination between the medical and surgical teams is needed, to improve the timing of the treatment. Indeed, patients who are further down on the waiting list will be invited to participate, in coordination with the Surgery Department. Moreover, additional measures are planned to improve motivation and acceptance of and adherence to the programme. More information about the group intervention will be given to patients when they are referred to our centre for bariatric surgery. An informative leaflet is being designed by the Endocrinology and Surgery Departments and will be explained and given to the patients in their first visit to them. The inclusion of this kind of approach in the routine care of patients with obesity should increase its acceptability. Alternative scheduling, or a combination of physical and online sessions, might also improve adherence in the case these measures are insufficient. These changes to the protocol will be submitted to the Ethics Committee.

The main strength of our study is the multidisciplinary approach of the intervention, which includes not only nutritional, behavioural, and psychological interventions, but also physical conditioning and respiratory training. The fact that it is designed as a randomized, controlled trial sets high expectations regarding the quality of the results. Once the SARS-CoV-2 allows, the clinical trial will start.

## 5. Conclusions

A randomized clinical trial protocol is described, which, for the first time, evaluates the effect of a preoperative physical and respiratory training programme, added to standard group intervention, including education and cognitive-behavioural therapy, in patients scheduled for bariatric surgery. The pilot study has uncovered difficulties in recruitment, adherence, and coordination, which will lead to the implementation of measures to solve them. The efficacy of the intervention will be assessed once the trial is carried out. If significant benefits are proved, this trial could lead to a paradigm shift in the pre-operative management of these patients.

## Figures and Tables

**Table 1:** Programme contents.

Session	Description	Contents
Session 1	Welcome and introduction	Introduction Questionnaires completion Group dynamics activity
	Medical aspects 1 (Endocrinologist)	Obesity and its complications Treatment: drugs, intragastric baloon How to prepare for surgery: healthy lifestyle, weight-loss, cardiovascular risk factors. Evidence Bariatric surgery: procedures. Benefits and risks. What to expect from surgery Physical activity. How to monitor
Session 2	Nutrition 1 (Dietician)	Food groups The Mediterranean diet as example of a healthy diet. Portion size Calories
Session 3	Psychoeducation 1 (Psychologist)	I am not fat. Differences between trait and state Weight regain and how to deal with it. The importance of cognitive-behavioural changes Personality and food Behavioural strategies with food: the importance of how and what to eat How to create a habit. Setting realistic goals
Session 4	Nutrition 2 (Dietician)	How to do the shopping Reading food labels
Session 5	Psychoeducation 2 (Psychologist)	Cognitive restructuring: identifying, analysing and modifying negative and irrational thoughts associated with eating behaviours How to say NO Self-control and travelling, celebrations, and eating out
Session 6	Nutrition 3 (Dietician)	Unnecessary foods Sweeteners Miraculous diets Myths
Session 7	Psychoeducation 3 (Psychologist)	Self-esteem and self-trust Strategies to reduce stress and anxiety Progressive relaxation technique
Session 8	Medical aspects 2 (Endocrinologist)	Postoperative diet. Protein sources. How to enrich food. Vitamin supplementation. Postoperative follow-up Weight regain When to go to the emergency room Changes in drug treatments after surgery. Long-term complications. Frequent problems after surgery: heartburn, reflux, dumping, diarrhoea. Skin flaps Pregnancy

**Table 2:** Baseline characteristics of the pilot study population, according to the assigned treatment group ^1^.

	Intervention (*n*= 7)	Control (*n*= 8)
Age (years)	38 ± 10	41 ±10
Sex (*n*) Women/Men	7/0	7/1
Weight (kg)	112.9 ±19.5	135.9 ±23.2
BMI (kg/m^2^)	44.7 ± 4.5	48.5 ± 6.7
Waist circumference (cm)	130.4 ± 12.1	138.0 ± 11.4
FM (%)	47.7 ± 4.1	52.4 ± 3.8
PA (°)	6.6 ± 0.6	6.0 ± 0.6
6MWD (m)	447.1 ± 83.5	433.5 ± 37.4
6MWD (%)	86.1 ± 16.1	90.8 ± 12.5
HGS Right Hand (kg)	24.0 ± 7.7	29.7 ± 9.1
HGS Left Hand (kg)	24.1 ± 2.6	26.2 ± 8.6
IPAQ ^2^		
Low	1 (20)	1 (16.6)
Moderate	2 (40)	5 (83.3)
High	2 (40)	0 (0)
NA	2	2
HbA1c (%)	5.9 ± 1.7	5.5 ± 0.4
Diabetes mellitus ^2^	3 (42.9%)	1 (12.5%)
Hypertension ^2^	3 (42.9%)	2 (25.0%)
HDL-cholesterol (mg/dL)	47 ± 10	41 ± 7
LDL-cholesterol (mg/dL)	114 ± 46	105 ± 25
Dyslipidaemia ^2^	2 (28.6%)	2 (25%)
NAFLD ^2^	1 (14.3%)	2 (25%)
Ferriman-Gallwey Score	0 ± 1	1 ± 1
Infertility ^2^	3 (42.9)	2 (25%)
Chronic venous insufficiency ^2^	2 (28.6)	2 (25%)
OSA ^2^	3 (75)	3 (50)
Function limiting osteoarthritis ^2^	2 (28.6)	4 (50%)
Gastroesophageal reflux/Hiatal hernia ^2^	1 (14.3)	1 (12.5%)
Bronchial asthma ^2^	2 (28.6)	3 (37.5%)
Intracranial hypertension ^2^	1 (14.3)	0 (0%)
EQ-5D-5L score	57 ± 23	47 ± 24
MEDAS		
High	1 (20)	7 (100)
Low	4 (80)	0 (0)
NA	2	1
EDI-3 score	168 ± 27	198 ± 49
HADS score	16 (7)	16 (5)
HADS ^2^		
Normal	3 (60)	1 (20)
Probable case	2 (40)	5 (80)
NA	2	2

^1^ Plus-minus values are the means ± SD. ^2^ Values are *n* (percentage of sample). BMI: body mass index; NA: not available; FM%: (fat mass percentage); PA: phase angle; 6MWD: six-minute walk distance; 6MWD: percentage of theoretical six-minute walk distance; HGS: handgrip strength; IPAQ: International Physical Activity Questionnaire; NAFLD: nonalcoholic fatty liver disease; OSA: Obstructive Sleep Apnea; EQ-5D-5L: EuroQol-5D-5L; MEDAS: Adherence to the Mediterranean Diet Adherence Screener; EDI-3: Eating Disorder Inventory; HADS: Hospital Anxiety and Depression Scale.

## Data Availability

The data presented in this study are available on request from the corresponding author. The data are not publicly available due to ongoing trial.
